# *Pdgfrα* deficiency in islet β-cells up-regulates apoptosis of beta-cells and disturbs glucose metabolism in B6 mice

**DOI:** 10.3389/fendo.2025.1630979

**Published:** 2025-10-29

**Authors:** Luyao Zhang, Yanpeng Xing, Pai Wang, Jianlei Gu, Jian Peng, Juan Huang, James Alexander Pearson, Youjia Hu, Hongyu Zhao, F. Susan Wong, Li Wen

**Affiliations:** ^1^ Department of Gastrocolorectal Surgery, General Surgery Center, The First Hospital of Jilin University, Changchun, Jilin, China; ^2^ Section of Endocrinology, Department of Internal Medicine, School of Medicine, Yale University, New Haven, CT, United States; ^3^ Department of Biostatiscs & Data Science, Yale School of Public Health, New Haven, CT, United States; ^4^ National Clinical Research Center for Metabolic Diseases, Key Laboratory of Diabetes Immunology (Central South University), Ministry of Education, Changsha, Hunan, China; ^5^ Division of Infection and Immunity, School of Medicine, Cardiff University, Cardiff, United Kingdom

**Keywords:** obesity, pancreatic beta cells, PDGFR alpha, apoptosis, ATF5, *Gadd45b*

## Abstract

**Introduction:**

Pancreatic β-cell dysfunction is a key contributor to the development of Type 2 Diabetes. The platelet-derived growth factor receptor α (PDGFRα) is known to play a crucial role in β-cell proliferation and expansion. However, its specific role in β-cell function and glucose metabolism remains unclear. This study aimed to investigate the effects of Pdgfrα deficiency on islet β-cell function and overall glucose metabolism.

**Methods:**

To explore this, we generated β-cell-specific Pdgfrα-deficient C57BL/6 mice (Pdgfra^fl/fl^ Pdx1-cre^+^) and assessed their metabolic function under both normal and high-fat diet conditions. Various parameters were measured, including body weight, body fat composition, glucose metabolism, insulin content, and β-cell apoptosis. Additionally, we conducted mechanistic analyses to understand the signaling pathways involved.

**Results:**

Pdgfrα-deficient mice exhibited significantly greater weight gain and increased body fat compared to controls. These mice also showed impaired glucose metabolism, reduced insulin content in β-cells, and increased β-cell apoptosis. Mechanistic studies revealed that Pdgfrα deletion led to suppression of Atf5 expression via downregulation of the PI3K pathway. This suppression resulted in enhanced β-cell apoptosis. Furthermore, Atf5 was found to regulate the expression of Gadd45b, Bcl2, and aminoacyl-tRNA synthetases, which are involved in insulin biosynthesis and glucose metabolism.

**Discussion:**

Our findings demonstrate that PDGFRα plays a critical role in maintaining β-cell function and glucose homeostasis. Loss of PDGFRα impairs β-cell survival and insulin production, likely through the PI3K–Atf5 axis. These insights suggest that targeting β-cell apoptotic pathways, particularly involving Atf5 and its downstream effectors, may offer promising avenues for the prevention and treatment of Type 2 Diabetes.

## Introduction

There are 537 million people living with diabetes worldwide, approximately 90% of whom have type 2 diabetes (1). Type 2 diabetes is characterized by insulin resistance in target organs and relative insulin deficiency due to pancreatic β-cell dysfunction and/or β-cell loss (2); however, the mechanisms by which β-cell dysfunction and β-cell loss are not fully understood. Studies have shown that glucotoxicity and lipotoxicity in type 2 diabetes, individually and together, impair β-cell function and/or damage β-cells (3). Glucotoxicity and lipotoxicity further lead to endoplasmic reticulum (ER) stress (4, 5), oxidative metabolism (6), and amyloid deposition (7), all of which can lead to β-cell dysfunction and loss of β-cell mass (8, 9). When the islets cannot sustain β-cell compensation for insulin resistance, blood glucose can no longer be adequately regulated, and type 2 diabetes is diagnosed.

Platelet-derived growth factor receptor (PDGFR) is a transmembrane receptor tyrosine kinase consisting of an extracellular ligand binding domain and an intracellular tyrosine kinase domain, which binds homodimers of PDGF-A, PDGF-B, PDGF-C, PDGF-D and heterodimer PDGF-AB (10–12). It engages with several well-characterized signaling pathways, that include Ras-MAPK, PI3K, PLC-γ, and various signaling molecules including enzymes, adaptors, and transcription factors (13, 14). PDGFR plays an important role in diverse cellular processes including the cell cycle, cell migration, cell metabolism and survival, as well as cell proliferation and differentiation (15). Pdgfrα is also involved in age-dependent β-cell proliferation and expansion (16). In aging mice, increased PDGF-AA level in the circulation promoted β-cell proliferation and function resulting in better glucose tolerance (17). In high fat diet-induced obese (HFDIO) mice, islet macrophages promote β-cell proliferation via a PDGFR signaling-dependent mechanism (18). In addition, miRNAs directly targeting Pdgfrα in β-cells resulted in the inability to proliferate in response to mitotic stimuli (19, 20).

We previously found that TLR9 deficiency upregulated PDGFRα gene expression and promoted pancreatic islet development as well as β-cell differentiation (21). To determine the effect of PDGFRα in modulating islet β-cell functions and glucose metabolism, we generated islet β-cell specific *Pdgfra*-deficient C57BL/6 mice (*Pdgfra^fl/fl^ Pdx1-Cre^+^* C57BL/6) with the littermate *Pdgfra^fl/fl^ Pdx1-Cre^-^* C57BL/6 mice used as controls. We found that Pdgfrα deficiency in pancreatic β-cells led to significant increase in the body weight, enhanced insulin resistance and reduced glucose tolerance in the *Pdgfra* ^fl/fl^ *Pdx1-Cre^+^* C57BL/6 mice. Furthermore, *Pdgfra* deficiency in pancreatic β-cells resulted in reduction of islet numbers and function by up regulating β-cell apoptosis, which was mediated by down regulation of *Atf5*, a pro-survival transcription factor.

## Methods

### Mice

Mice used in the study were housed in strict specific pathogen-free (SPF) facilities with a 12-hour-dark/light cycle in the Yale Animal Resource Center (YARC). *Pdgfra* ^fl/fl^ *C57BL/6* breeders were kindly provided by Valerie Horsley (Yale University) and *Pdx1-cre^+^ C57BL/6* breeders were kindly provided by Qingchun Tong (UT Health). We bred the two mouse lines and generated *Pdgfra^fl/fl^Pdx1-cre^+^ C57BL/6* and *Pdgfra^fl/fl^Pdx1-cre^-^ C57BL/6* littermates for the study. The mice were fed with either autoclaved normal diet (Teklab Global, USA, 6.2% fat) or high fat diet (HFD, Research Diet, 60% fat, New Brunswick, NJ, USA) ad libitum. The use of the animals in this study was approved by the Institutional Animal Care and Use Committee of Yale University.

### Cell line

NIT-1 (ATCC CRL-2055), a mouse β-cell line, was purchased from ATCC (Manassas, VA, USA.). NIT-1 cells have well developed rough endoplasmic reticulum and β-granules with similar ultrastructural features to differentiated mouse β-cells (43). They were cultured in Ham’s F12K medium with 2 mM L-glutamine, 1.5 g/L sodium bicarbonate (ATCC), and 10% heat-inactivated fetal calf serum (FCS (Gemini)).

### Antibodies and reagents

Most of the fluorochrome-conjugated monoclonal antibodies used in this study were purchased from BioLegend unless otherwise stated. The supernatants of different monoclonal antibody (mAb) hybridomas were provided by the late Charles Janeway (Yale University). RPMI-1640 medium and heat-inactivated FCS were purchased from Invitrogen and Gemini respectively. Anti-H2-K^d^ mAb, was affinity purified from hybridoma (clone: HB159) supernatant. Anti-Qa-2 mAb was purchased from BioLegend (clone: 659H1-9-9). The lysis buffer and other reagents for Western blot (WB) were purchased from ThermoFisher and Bio-Rad. Rabbit anti-mouse antibodies for WB were purchased from ThermoFisher and Cell Signaling.

### Intra-peritoneal glucose tolerance test

Mice were fasted overnight with free access to water and intra-peritoneal (i.p.) glucose tolerance tests (IPGTTs) were performed by i.p. injection of glucose (1 g/kg to the mice fed with high fat diet and 2 g/kg to the mice fed with normal diet). The blood glucose was measured with a FreeStyle glucose meter (Abbott) before (time zero) and at different time points after glucose injection.

### Insulin tolerance test

Mice were fasted for 4 h with free access to water and insulin tolerance tests (ITTs) were performed by i.p. injection of insulin (Humulin-R, 0.75 U/kg; Eli Lilly, Indianapolis, IN, USA). The blood glucose was measured with a FreeStyle glucose meter (Abbott) before (time zero) and at different time points after insulin injection.

### Islet isolation

Pancreatic islets were isolated as described (44). Briefly, mice were sacrificed by cervical dislocation before dissection. The pancreas was inflated through the bile duct with a 30G needle starting at the gall bladder with 3 ml cold collagenase (Sigma; St Louis, MO, USA) solution (0.3 mg/ml). The pancreas was dissected, weighed and placed into a siliconized glass vial containing 1 ml of 1 mg/ml collagenase solution. The sealed vials were incubated in a 37°C water bath for 12–14 min with vibration. After three washes of the digested pancreas, islets were handpicked and counted under a dissecting microscope for further experiments.

### Insulin content assay

The insulin content measurement was performed as previously described (45). Briefly, the middle part of the pancreatic body was dissected, weighed and placed into an Eppendorf tube with 0.5ml ice-cold acid-ethanol buffer (1.5% concentrated HCL, 75% Ethanol and 23.5% deionized water) and homogenized by an Ultrasonic Homogenizer (VWR Scientific, Radnor, PA, USA) for 2 min with 20 pulses. After further incubating the mixture at 4°C overnight, the homogenized tissue was centrifuged at 1400g for 20 min at 4°C. Insulin content in the supernatant was measured using an insulin RIA kit (EMD-Millipore, Burlington, ME, USA).

### Insulin release assay

The insulin release assay was performed as previously described (21). Briefly, isolated pancreatic islets were mixed and equally distributed to test tubes (30–60 islets/tube depending on the total islet numbers) after stabilizing in low-glucose KRB buffer for 2 hours. The islets were then stimulated with KRB containing high glucose (25 mmol/l) and the supernatant fractions were harvested every 5 min after glucose stimulation. Secreted insulin in the supernatant fractions was measured using the insulin RIA kit (EMD-Millipore, Burlington, ME, USA).

### Evaluation of islet mass

Ex vivo pancreases from 18-22-week-old male mice were fixed in periodate–lysine–paraformaldehyde followed by freezing in Tissue-Tek OCT (Bayer, Elkhart, IN, USA). Each pancreas was cut in its entirety into more than two hundred 10 μm thick sections and one section was selected at a 9-section interval for staining with hematoxylin to ensure different islets were imaged. Islet mass was measured using Image J software (NIH, Bethesda, MD, USA) after photographing under the light microscope.

### Quantitative PCR

RNA from pancreatic islets isolated as described above was extracted with RNeasy Mini Kit (Qiagen, Hilden, Germany) and quantified by NanoDrop (ThermoFisher). Equal amounts of RNA were reverse transcribed using iScript cDNA Synthesis Kit (Bio-Rad, Hercules, CA, USA). Quantitative PCR (qPCR) was performed using the Bio-Rad iQ5 qPCR detection system (Hercules, CA, USA) with the specific primers (**Supplementary Table S2**). The level of gene expression was determined with the 2−ΔΔCt method by normalization with the reference gene *gapdh*.

### Cell staining and flow cytometry analysis

The isolated islets were treated with Cell Dissociation Solution (Sigma) to obtain the single-cell suspension. Cells were stained with fluorochrome-conjugated monoclonal antibodies to CD45 (for immune cells, BioLegend; San Diego, CA, USA), CD140a (for all cells, BioLegend) and FluoZin-3-acetoxymethyl (for β-cells, ThermoFisher, Waltham, ME, USA) (46). The β-cell survival status was assessed with APC Annexin V Apoptosis Detection Kit with 7-AAD (BioLegend) following the manufacturer’s protocol. Cells were analyzed on a BD LSRII flow cytometer (LSRII; BD Bioscience, San Diego, CA, USA) and results were analyzed with FlowJo 10.4 software.

### RNA-sequencing and analysis

RNA from pancreatic islets isolated as described above was extracted with RNeasy Mini Kit (Qiagen, Hilden, Germany) and quantified by NanoDrop (ThermoFisher). RNA-sequencing (poly A) was performed at the Yale Center for Genome Analysis using NovaSeq with HiSeq paired-end, 100bp. The raw TRAPseq fastq files were processed using the fastp tool (version 0.20.0) (47). With a default setting, sequencing reads with low-quality bases were trimmed or filtered. Alignment was performed for cleaned reads using STAR (version 2.7.9) (48) and mouse reference genome (gencode version GRCm38.p6 with vM25 gene annotation). Expression quantification was performed for alignment results using featureCounts (version 2.0.0) (49). As genes with low expression levels that represent noise were to be excluded before downstream analysis, we defined low expression filtering as expressed genes with ≥ 6 read counts in at least 20% of samples. The filtered read counts matrix was then normalized by transcripts per million (TPM) method. Detection of differentially-expressed genes was performed using R package DESeq2 (version 1.30.1) (50). The Benjamini-Hochberg procedure was used for multiple test correction, and FDR ≤ 0.05 as a threshold for detection of differentially expressed genes.

### Western blotting

Western blot (WB) analysis was performed as previously described (51). The isolated islets (100) were homogenized in 150μl lysis buffer RIPA (ThermoFisher Scientific, Pittsburgh, PA) with phosphatase and EDTA inhibitor (Roche, Mannheim, Germany). Total protein concentrations were quantified using the BCA assay (Pierce, Rockford, IL). Ten micrograms of total protein were dissolved in 2× loading buffer (Bio-Rad) and separated on a MINI-PROTEAN TGX Stain-Free Gels (4-20%, 10 well, 30μl) (Bio-Rad, Hercules, CA, USA). The proteins were then transferred to polyvinylidene fluoride membranes (Bio-Rad). Membranes were blocked with 5% nonfat milk (ThermoFisher Scientific, Pittsburgh, PA) and incubated overnight with primary antibodies (**Supplementary Table S3**) in SDS buffer (Bio-Rad). Membranes were then washed and incubated with the appropriate horseradish peroxidase-conjugated secondary antibodies (BioLegend) at room temperature for 2 h. After washing, membranes were incubated with West Pico Plus (ThermoFisher Scientific, Pittsburgh, PA) to visualize the proteins.

### Data analysis

Statistical analysis was performed using GraphPad Prism software version 9.0 (GraphPad Software, San Diego, CA, US). Islet mass was analyzed using a Kolmogorov-Smirnov test. Data from other assays were analyzed with either a two-tailed Student’s t test (if data normally distributed), a two-tailed Mann-Whitney test (if data not normally distributed), multiple t tests with FDR correction, or a two-way ANOVA. P < 0.05 was considered to be significant.

## Results

### Effects of Pdgfrα deficiency in pancreatic β-cells on metabolism in C57BL/6 mice

**Figure 1:**
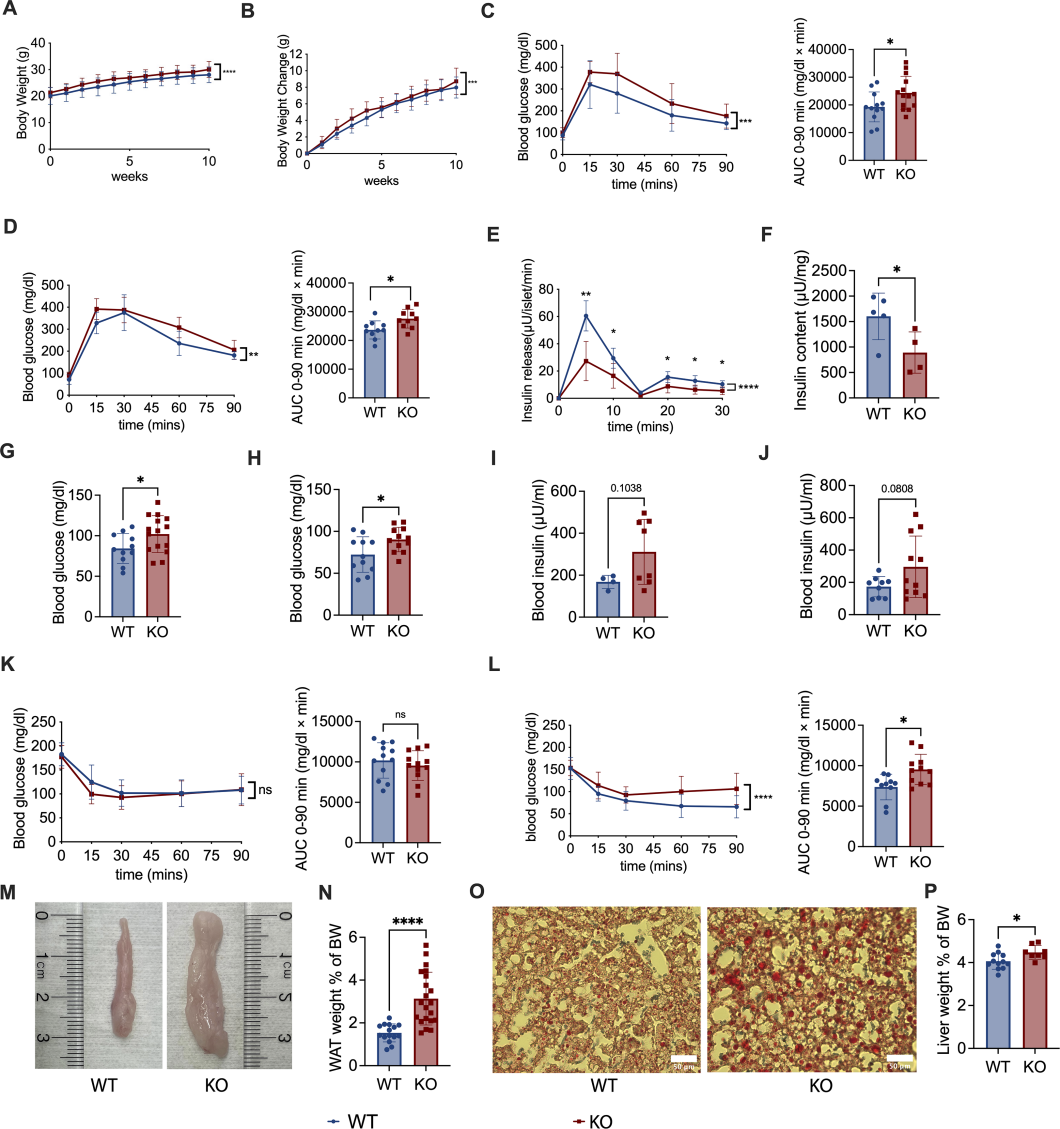
*Pdgfrα* deficiency in pancreatic β-cells impairs metabolism in C57BL/6 mice fed with normal diet. **(A)** Body weight change of *Pdgfrα^fl/fl^ Pdx1-Cre* ^+^ (KO) mice and *Pdgfrα^fl/fl^ Pdx1-Cre* ^-^ control mice (n=12/group). Weekly body weight measurements were started in 6-wk-old mice shown in the figures as week 0. **(B)** Net body weight gain of KO mice and control mice (n=12/group). Body weight change was calculated by subtracting the initial body weight (6-wk-old) from the measured body weight. **(C, D)** IPGTT results and the AUC of KO mice and control mice respectively, at 6 weeks old **(C)** (n=11-15), **(D)** 14 weeks old (n=10-11). **(E)** Insulin release assay of islets from KO mice and control mice at ~ 18 weeks old (n=5/group). **(F)** Insulin content of pancreas from KO mice and control mice at ~18 weeks old (n=5/group). **(G, H)** Fasting blood glucose of KO mice and control mice at 7 weeks old **(G)** (n=10-15) and **(H)** ~20 weeks old (n=11-12). **(I, J)** Fasting blood insulin of KO mice and control mice at 7 weeks old **(I)** (n=4-8) and ~20 weeks old **(J)** (n=9-11) **(K, L)** ITT results of KO mice and control mice at 7 weeks old **(K)** (n=12-13), 15 weeks old **(L)** (n=10-11), and the AUC, respectively. **(M)** Epididymal fat, representative of white adipose tissue (WAT), shown from KO mice and control mice. **(N)** Percentage of WAT weight to body weight (BW) of KO mice and control mice (n=15-21). The percentage of WAT weight to BW was calculated by dividing the bilateral epididymal fat weight by the body weight. **(O)** Representative liver sections after staining with oil red O for fat droplets are shown. Scale bar, 50 μm. **(P)** Percentage of liver weight to BW of KO mice and control mice (n=6/group). All the data were pooled from at least two independent experiments. Male mice were used in the experiments. A-E, K & L were analyzed by two-way ANOVA, C, D, F-J, N & P were analyzed by two-tailed Student’s *t*-test, and L was analyzed by two-tailed Mann-Whitney test. The variations are represented as mean ± SD or median ± 95%CI, respectively. *p < 0.05, ****p < 0.0001.

*Pdgfra* plays a role in age-dependent β-cell proliferation and expansion (16), but how Pdgfrα affects islet β-cell function and glucose metabolism in obesity and type 2 diabetes was previously unknown. To study this, we generated *Pdgfra^fl/fl^ Pdx1-Cre^+^* C57BL/6 mice (KO mice) and *Pdgfra^fl/fl^ Pdx1-Cre^−^* C57BL/6 littermates (WT mice). We confirmed that Pdgfrα protein (CD140a) was reduced in islet β-cells by flow cytometry (**Supplementary Figure S1A**). We also examined the expression of CD140a at different mouse ages - young (3–4 wks) and adults (10–12 wks). As expected, KO mice have lower CD140a expression (**Supplementary Figure S1B**); however, it is interesting that the difference between KO and control mice was much greater in young mice, and the proportion of CD140a-expressing islet β-cells is also higher (over 10%, see Y axis) and less than 1% (see Y axis) in adult KO and control mice respectively (**Supplementary Figure S1B**). We first monitored the body weight (BW) of the KO and WT male mice fed with a normal diet (**Supplementary Figure S2**) and observed that the KO mice gained more body weight (**Figures 1A,B**) compared to the control mice on normal diet, although there was no difference in food intake (**Supplementary Figure S3**). Next, we assessed glucose metabolism in KO mice and control mice, fed with normal diet by performing IPGTT at different ages (6–7 and 14 weeks), and KO mice in both age groups exhibited impaired glucose tolerance (**Figures 1C,D**). To further evaluate the secreted/synthesized insulin by β-cells in pancreatic islets, we performed insulin release assays and found that islets from KO mice secreted less insulin compared to the control mice (**Figure 1E**) and that the pancreata from KO mice contained less insulin (**Figure 1F**). In addition, both age groups of KO mice had higher fasting blood glucose compared to the age-matched control mice (**Figures 1G,H**). Interestingly, although KO mice secreted less insulin, we found that there was a trend to higher fasting blood insulin levels in KO mice, but these differences between KO and control mice were not statistically significant at any age tested (**Figures 1I,J**).

To assess insulin resistance, we performed insulin tolerance tests (ITT) and found that there was no significant difference between adolescent KO and control mice (**Figure 1K**), but the adult KO mice showed pronounced insulin resistance (**Figure 1L**). This suggests that although decreased insulin secretion was present from an early age, the insulin resistance was age-related in our model system. In line with the body weight, KO mice fed with normal diet had more abdominal fat including epididymal fat, (**Figure 1M**), with the ratio of white adipose tissue (WAT), calculated using epididymal fat to body weight, also significantly higher in KO mice, compared to the control mice (**Figure 1N**). Moreover, we found more fat in the liver of KO mice fed with normal diet than WT mice (**Figure 1O**), and there was a higher ratio of liver weight to body weight in the KO mice than the control mice (**Figure 1P**).

To assess if *Pdgfra* deficiency in pancreatic islet β-cells affects islet architecture and the composition of islet cells, especially islet α cells that also regulate blood glucose, we performed pancreatic histology and histochemical staining of KO and control mice. We found that the size of the islets from the KO mice were generally smaller than in control mice but there was no alteration of islet architecture (**Supplementary Figure S4A**). Supporting our insulin release data (**Figure 1E**), the staining of insulin in the islets of KO mice was weaker than in control islets (**Supplementary Figure S4B**). Interestingly, it appears that there were more glucagon-positive α cells in the islets of KO mice, compared to the control mice (**Supplementary Figure S4C**); however, as the islets from the control mice were generally larger than those from the KO mice, it is conceivable that the “absolute” number of islet α cells was comparable between the KO and control mice. Thus, our results demonstrate that *Pdgfra* deficiency in pancreatic islet β-cells results in significantly impaired glucose metabolism, increased body weight, body fat and fatty liver in C57BL/6 mice fed with normal chow.

### High fat diet (HFD) exacerbates metabolic dysregulation in C57BL/6 mice with Pdgfrα deficiency in pancreatic β-cells

**Figure 2:**
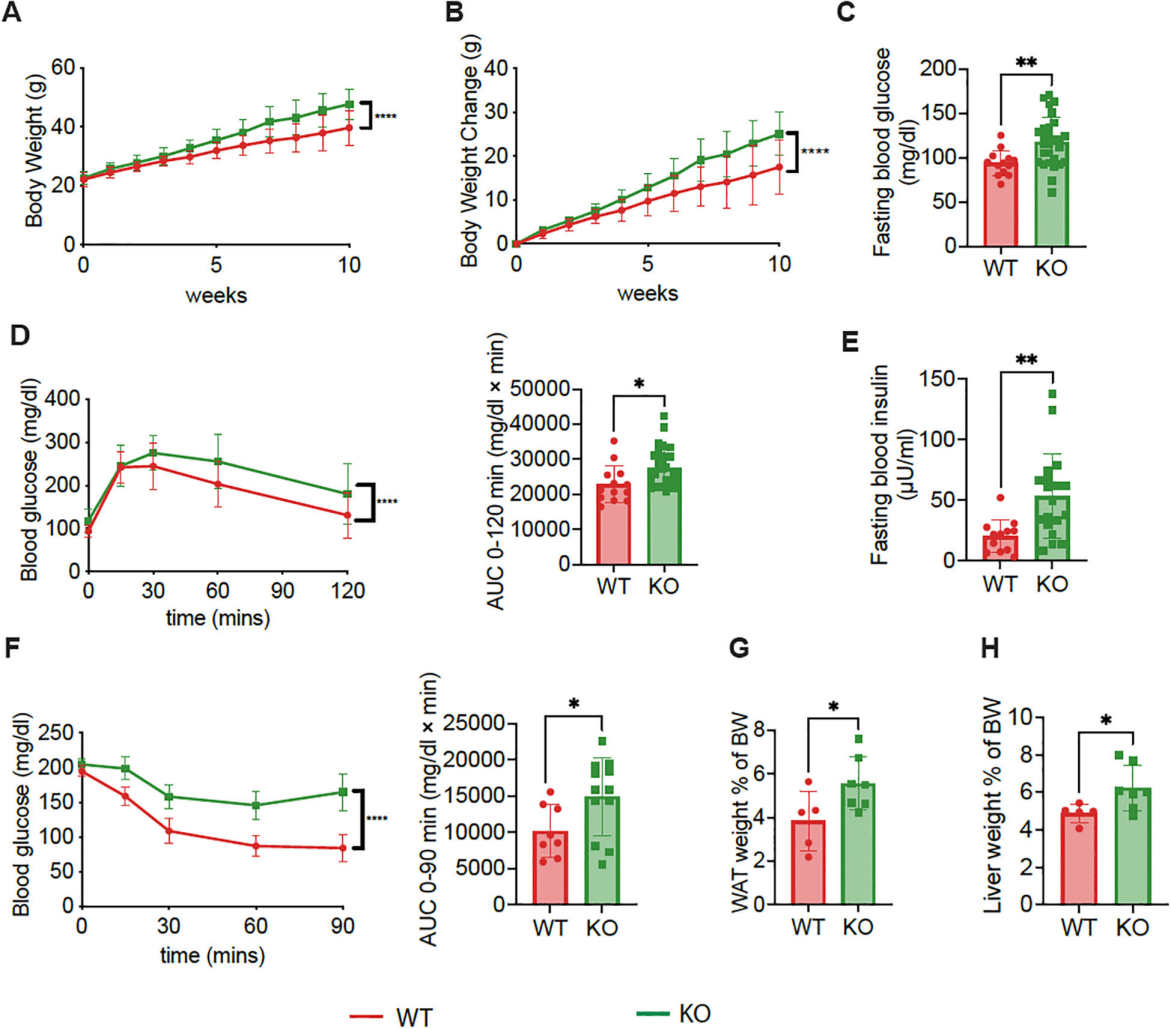
Exacerbated metabolic dysregulation in C57BL/6 mice with *Pdgfrα*-deficiency in β-cells fed with HFD. **(A)** Body weight change of KO mice and control mice during 10-weeks of HFD, which was started at 6 weeks of age (n=9-10). **(B)** Net body weight gain of KO mice and control mice (n=9-10). **(C)** Fasting blood glucose of KO mice and control mice at 14 weeks of age after 8 weeks of HFD (n=13-28). **(D)** IPGTT results of KO mice and control mice after 8 weeks of HFD (n=13-28, 14 weeks of age), and AUC are shown. **(E)** Fasting blood insulin of KO mice and control mice at ~20 weeks of age (n=12-19). **(F)** ITT results of KO mice and control mice at 15 weeks of age (n=8-12), and AUC are shown. **(G)** Percentage of WAT weight to BW of KO mice and control mice (n=5-7). The percentage was calculated as described (**Figure 1N**). **(H)** Percentage of liver weight to BW of KO mice and control mice (n=5-7). All the data were pooled from at least two independent experiments (male mice were used in the experiments) and analyzed by a two-way ANOVA in A-C & F, or by a two-tailed Student’s *t*-test in D, E, G & **(H)** The variations are represented as mean ± SD. *p<0.05, **p < 0.01, ****p < 0.0001.

To identify the impact of *Pdx*-Cre-driven *Pdgfra* deficiency on glucose metabolism in a metabolically stressed condition, we fed 6-week-old KO and control mice with high fat diet (HFD) for 8 weeks (**Supplementary Figure S5**). The body weight gain in the KO mice was greater compared to the control mice (**Figures 2A, B**) and the KO mice had much higher fasting blood glucose and significantly worse glucose tolerance, assessed by IPGTT, compared to control mice (**Figures 2C, D**). Fasting blood insulin levels were also significantly higher in KO mice than in the control mice (**Figure 2E**). In line with the KO mice on normal diet (**Figure 1L**), but more pronounced, the KO mice on HFD had higher insulin resistance, assessed by ITT, compared to the control mice (**Figure 2F**). Given the fact that KO mice had decreased insulin synthesis and/or secretion (**Figures 1E, F**), this suggested that insulin resistance was, in fact, even more severe. Furthermore, we found that the ratio of white adipose tissue (WAT) to body weight and the ratio of liver to body weight in KO mice were significantly higher, compared to the control mice (**Figures 2G, H**). Taken together, our results from high fat diet-fed mice agreed with those from normal chow-fed mice, indicating that high-fat diet exacerbated the metabolic dysfunction.

### Increased skeletal muscle inflammation in C57BL/6 mice with Pdgfrα deficiency in pancreatic β-cells

**Figure 3:**
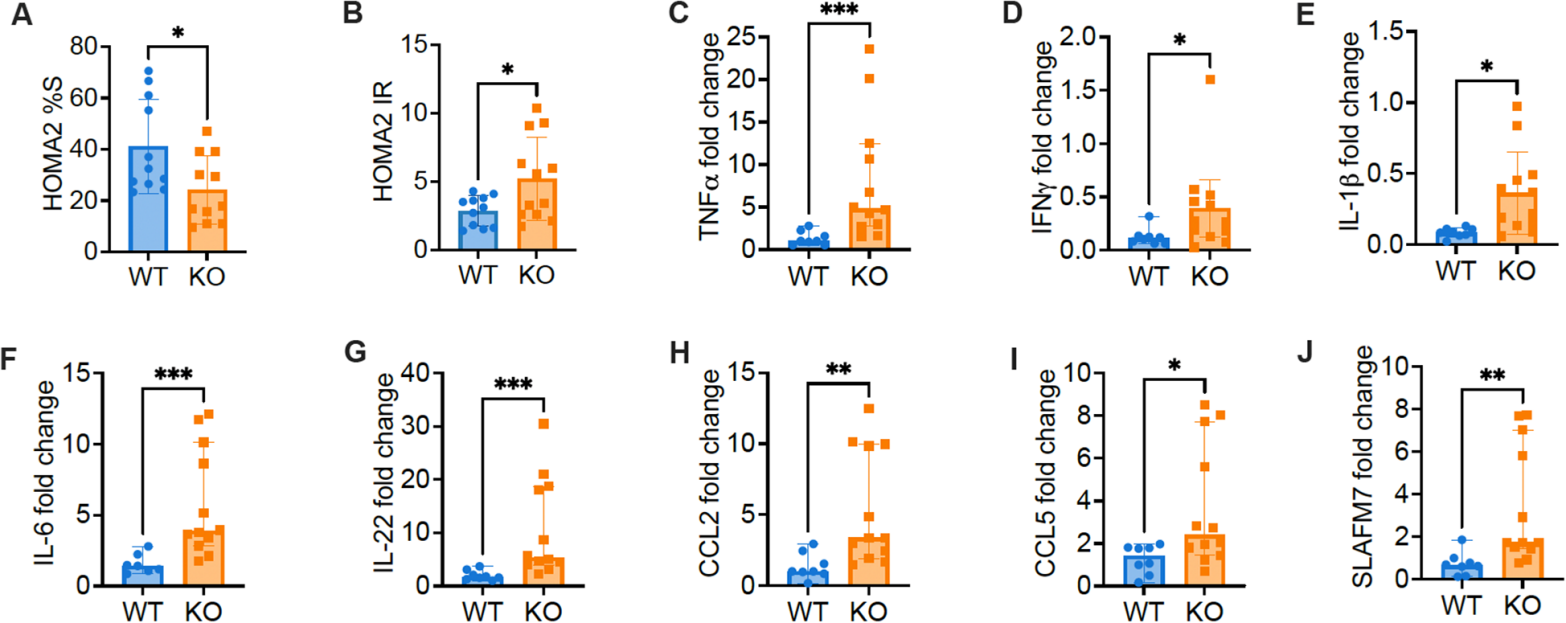
The expression of inflammatory factors and chemokines increase in skeletal muscle of C57BL/6 mice with *Pdgfra* deficiency in β-cells. Insulin resistance was estimated with the Homeostasis Model Assessment 2 (HOMA2). **(A)** HOMA2%S and **(B)** HOMA IR were calculated and data from KO mice and control mice fed with normal food. Skeletal muscle RNA was extracted from 18-22-week-old male KO mice and control mice fed with normal food and qPCR was performed with specific primers. *Gapdh* was utilized as the internal reference gene for normalization. The gene expression level was calculated using 2^-ΔΔCT^ method. **(C)** TNFα (n=8-12), **(D)** IFNγ (n=8-12), **(E)** IL-1β (n=8-12), **(F)** IL-6 (n=8-12), **(G)** IL-22 (n=8-12), **(H)** CCL2 (n=8-12), **(I)** CCL5 (n=8-12), and **(J)** SLAFM7 (n=8-12). Data were pooled from two independent experiments (male mice were used in the experiments) and analyzed by a two-tailed Student’s *t*-test **(A, B, E)** and a two-tailed Mann-Whitney test **(C, D, F–J)**. The data are represented as mean ± SD or median ± 95% CI. *p < 0.05, **p < 0.01, ***p < 0.001.

As the KO mice without metabolic stress showed impaired metabolism and insulin resistance, to further verify the impaired beta cell function and insulin resistance, we focused on studies with normal diet. We calculated index of Homeostasis Model Assessment 2 (HOMA2-%S) and HOMA IR and found decreased insulin sensitivity but increased insulin resistance in KO mice fed with normal diet, compared to the control mice (**Figures 3A, B**). Skeletal muscles make up the most important organ for whole-body glucose homeostasis, and insulin resistance in skeletal muscle is an important feature of obesity and T2D (22). We assessed inflammation by qPCR, in skeletal muscle from KO mice fed with normal diet, compared to the control mice. Supporting the observation of insulin resistance in KO mice, we found higher expression of inflammatory cytokines, including TNFα, IFNγ, IL-1β, IL-6 and IL-22, in the muscles of KO mice fed with normal diet, compared to the control mice (**Figures 3C–G**). Furthermore, in keeping with the higher levels of inflammatory cytokines tested, higher expression of inflammatory chemokines such as CCL2, CCL5 and SLAFM7 were also found in the muscles of KO mice, compared to the controls (**Figures 3H–J**). Our results indicated that *Pdgfra* deficiency in pancreatic β-cells not only results in impaired β-cell function and glucose metabolism but also leads to heightened inflammation in muscle tissue, which likely contributes to the insulin resistance and body weight gain.

### Pdgfrα deficiency in β-cells reduces the islet mass and function, increases the β-cell apoptosis and alters Bcl2 expression in β-cells in C57BL/6 mice

**Figure 4:**
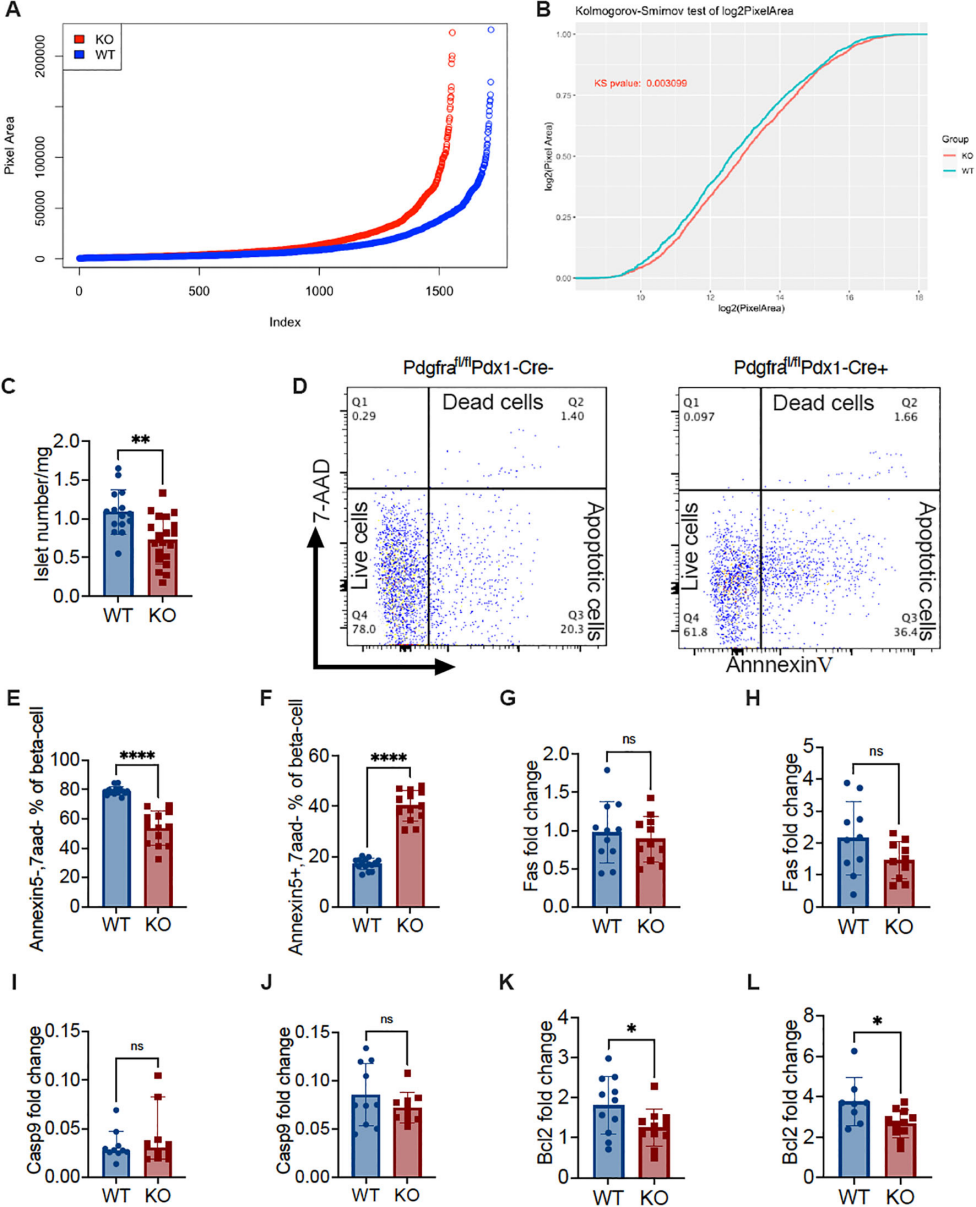
*Pdgfrα* deficiency decreases islet number and volume, increases β-cell apoptosis and alters Bcl2 expression in β-cells in C57BL/6 mice. **(A)** Ranking of islet number and area using ImageJ from approximately 600 sections from KO and control mice on a normal diet (n=3/group, ~200 section/mouse). **(B)** Kolmogorov-Smirnov test of **(A)**. **(C)** Isolated islet number from KO and control mice were standardized to the islet number per milligram of pancreas weight (n=15-20). **(D)** Representative FACS plots illustrating apoptosis of islet β-cells, identified by staining with FluoZin^+^ and CD45^-^, was determined by Annexin V and 7-AAD staining. **(E)** Live β-cells (Annexin V^-^/7-AAD^-^) (n=15-13); **(F)** apoptotic β-cells (Annexin V^+^/7-AAD^-^) (n=15-13). **(G, H)** *fas* gene expression levels of the islets from the mice fed with normal diet (G; n=11/group) or from the mice fed with HFD (H; n=10/group). **(I, J)** *caspase9* gene expression levels of the islets from mice fed with normal diet (I; n=10/group) or from mice fed with HFD (J; n=10/group). **(K, L**) *bcl2* gene expression levels of the islets from the mice fed with normal diet (K; n=11/group) or from the mice fed with HFD (L; n=8-11/group). Data were pooled from at least two independent experiments (male mice were used in the experiments) and analyzed by a two-tailed Student’s *t*-test in **(C, E-H, J-L)** or by a two-tailed Mann-Whitney test **(I)**. The variations are represented as mean ± SD or median ± 95%CI, respectively. *p < 0.05, **p < 0.01, ****p < 0.0001.

Next, we investigated whether *Pdx*-Cre-driven *Pdgfra* deficiency affected islet development and function in C57BL/6 mice. We first evaluated islet mass by measuring the islet area under light microscopy on pancreatic tissue sections of the entire pancreas from KO and control mice. We found that islet mass in KO mice was lower than in control mice using Image J analysis (**Figure 4A**). Kolmogorov-Smirnov test analysis indicated that there were significantly fewer islets and reduced islet area in the pancreata of KO mice compared with controls (**Figure 4B**). We also measured the net islet number in the same pancreatic weight of KO and control mice and analyzed the number of islets per milligram pancreas weight. We found fewer islets per milligram of pancreas in the KO mice, compared with the control mice, both of which were fed with normal diet and the experiments were performed in parallel (**Figure 4C**). The low islet number led us to hypothesize that β-cells in KO mice could be prone to apoptosis. To test our hypothesis, we performed Annexin V staining using ex vivo freshly prepared dispersed islet cells. Islet cells were co-stained with a β cell marker FluoZin and the immune cell marker CD45, in addition to Annexin V staining, prior to analyzing by flow cytometry, with β-cells gated as CD45 negative and FluoZin positive (CD45-FluoZin+). We found that there were more live β-cells (Annexin5-7AAD-) in control mice than in KO mice (**Figures 4D, E**), and conversely, more apoptotic β-cells (Annexin5 + 7AAD-) in KO mice than in control mice (**Figures 4D, F**). Thus, our data indicated that *Pdgfrα* deficiency in pancreatic β-cells enhanced β-cell apoptosis, which may contribute to the reduced islet number and function observed in the KO mice.

Next, we assessed pro-apoptotic (Fas and Caspase9) and anti-apoptotic (Bcl2) gene expression in the islets. To our surprise, there were no significant differences in the pro-apoptotic gene expression, regardless of the diet (**Figures 4G–J**). In contrast, the expression of the anti-apoptotic gene Bcl2 was significantly reduced in KO mice compared to the control mice, and this was diet independent (**Figures 4K, L**). This suggested that *Pdgfra* deficiency in islet β-cells did not increase pro-apoptotic signaling in β-cells but rather affected the ability of β-cells to survive.

Next, we evaluated islet β-cell proliferation, staining dispersed islet cells with Ki67, co-staining with anti-mouse CD45 and FluoZin and analyzing by flow cytometry. After gating islet β-cells (CD45-FluoZin+), we analyzed the proportion of Ki67 positive (Ki67+) cells. It is interesting that islets from KO mice had many fewer proliferating Ki67+ β-cells compared with control mice (**Supplementary Figure S6**).

### The role of Atf5 and Gadd45b in β-cells deficient in Pdgfrα

**Figure 5:**
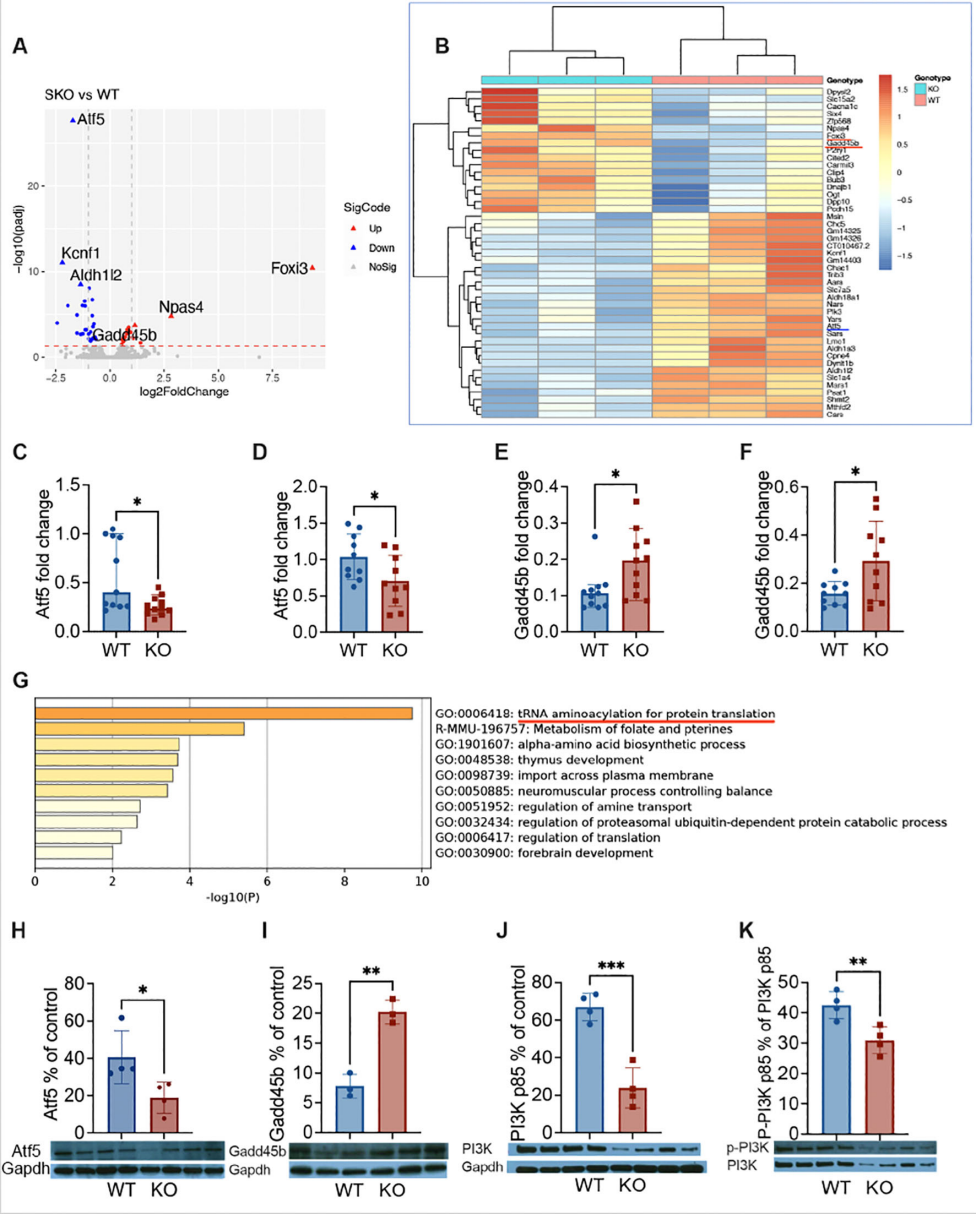
The expression of *atf5* and *gadd45b* in β-cells deficient in *Pdgfrα*. RNA sequencing analysis was performed on purified total RNA of islets from ~20-week-old male KO mice and control mice fed with normal diet. **(A)** Volcano plot, differential gene expression (DGE) analysis was performed to compare combined gene expression in islets from the two groups. Up (red) and Down (blue) represent up-regulated or down-regulated gene expressions respectively (adjusted p<0.05). **(B)** Heatmaps, Up and Down genes were selective, and clustered according to Gene Ontology analysis. To confirm the gene expression of *atf5* and *gadd45b* identified by RNA-seq, qPCR was performed from the islets of the male mice fed with HFD (~20 weeks of age). The relative gene expression levels were shown using 2−ΔΔCt method by normalization with the reference gene *gapdh*. **(C, D)** *atf5* gene expression levels from the mice fed with normal diet (C; n=11/group) or HFD (D; n=10/group). **(E, F)** *gadd45b* gene expression levels from the mice fed with normal diet (E; n=11/group) or HFD (F; n=10/group). **(G)** Top 8 clusters after pathway and process enrichment analysis with their representative enriched terms (one per cluster). **(H, I)** To confirm the expression of Atf5 and Gadd45b in islet at protein level, Western blotting was performed using islets from the KO and control mice fed with normal diet, bands were quantitated by densitometry and are presented as a percentage of control Gapdh values. **(H)** Atf5 protein expression level was measured by WB. **(I)** Gadd45b protein expression level was measured by WB. **(J, K)** The protein expression level of PI3K was also assessed. **(J)** PI3K p85 protein expression levels. **(K)** The P-PI3K p85 protein expression levels, which were expressed as a percentage of PI3K p85 values. Data were pooled from two independent experiments **(C-F)** and analyzed by a two-tailed Mann-Whitney test, and shown as median ± 95%CI. The experiments presented in **(H-K)** were performed twice and the results from one of the two experiments are shown. The data were analyzed by a two-tailed Student’s *t*-test and the results are shown as mean ± SD. *p < 0.05, **p < 0.01, ***p < 0.001.

To further investigate the molecular mechanism by which *Pdgfra* alters β-cell survival, we studied the transcriptome of the islets from KO and control mice by total RNA sequencing. The principal component analysis showed that the two groups of samples were clearly separated, indicating that the transcriptomes of the two groups of samples were very different (**Supplementary Figure S7A**). Transcriptome analysis showed that there were 45 genes expressed significantly differently between the two groups, of which 17 were upregulated, and 28 were downregulated in the islets from KO mice compared to the control mice (**Figures 5A, B**). We found that *atf5* (activating transcription factor 5) was the most downregulated gene while *gadd45b* (Growth Arrest and DNA Damage Inducible Beta), *Foxi3* (Forkhead box i3) and *Naps4* (neuronal PAS domain protein 4) were among the highest upregulated genes in the islets from KO mice (**Figures 5A, B**). The down-regulation of *atf5* and up-regulation of *gadd45b* were further confirmed by qPCR of the islets of mice fed with normal diet or HFD (**Figures 5C–F**; **Figures S7B, C)**. Interestingly, both *atf5* and *gadd45b* genes are associated with apoptosis caused by cellular stress. Atf5 is a cellular pro-survival transcription factor (23, 24) whereas Gadd45b is associated with apoptosis, often through Fas-mediated apoptosis (25). The gene enrichment analysis revealed that the most significantly enriched functional gene set was the tRNA aminoacylation for protein translation, which is also associated with ER stress (**Figure 5G**; **Supplementary Table S1**). Next, we assessed the levels of protein expression of Atf5 and Gadd45b in the islets by Western blot. Supporting the gene expression profile by RNA-seq and qPCR, Atf5 protein expression was significantly lower (**Figure 5H**), whereas Gadd45b protein expression was significantly higher (**Figure 5I**) in the islets of KO mice compared with the control mice. Atf5 expression is associated with PI3K (26, 27), which is an important intracellular kinase regulating many cell functions especially cell growth and survival (28). Interestingly, it is also a target of Pdgfrα (29, 30). Thus, we investigated the protein expression of PI3K by Western blot. Our results showed that the protein expression of PI3K regulatory subunit p85 and the phosphorylated P-PI3K p85 in the islets of KO mice were both significantly reduced compared to the islets from control mice (**Figures 5J, K**). Taken together, we found two altered genes, Atf5 and Gadd45b, which have not been reported before, contributing to β-cell apoptosis and survival in association with Pdgfrα through the PI3K pathway.

### Pdgfrα inhibitor promotes apoptosis upregulation and reduces insulin secretion in NIT-1 β-cells by regulating the expression of Atf5 and Gadd45b

**Figure 6:**
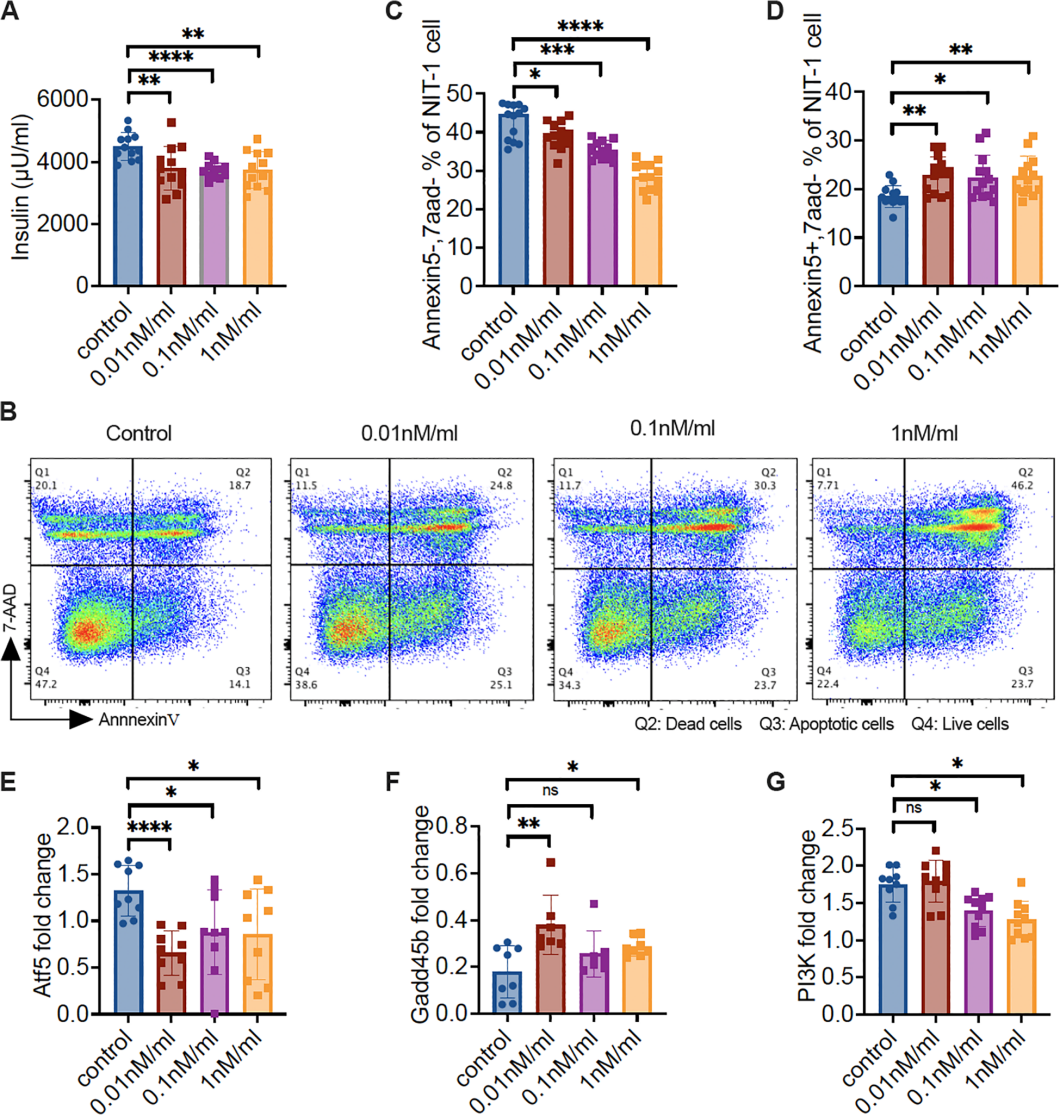
Pdgfrα inhibitor reduces the insulin secretion of NIT-1 cells, increases NIT-1 cell apoptosis, and alters the gene expression profile. NIT-1 cells were cultured till confluence, followed by treatment with different concentrations of Pdgfr inhibitor (CP673451) for 12 hours. The supernatant was collected for insulin measurement and cells were collected for flow cytometry staining and RNA extraction. **(A)** Insulin concentration of the supernatant from gradient Pdgfr inhibitor-treated NIT-1 cells. **(B)** Representative FACS plots are shown. Apoptosis of the gradient Pdgfr inhibitor treated NIT-1 cells were determined by Annexin V and 7-AAD staining, gated on the single cells. **(C)** Live NIT-1 cells (Annexin V^−^/7-AAD^−^) (n=13/group); **(D)** Apoptotic NIT-1 cells (Annexin V^+^/7-AAD^−^) (n=13/group); **(E-G)** The qPCR was performed with the RNA from gradient Pdgfr inhibitor-treated NIT-1 cells. **(E)** *atf5* gene expression levels (n=9/concentration), **(F)** *gadd45b* gene expression levels (n=7-8/concentration) and **(G)** *pi3k* gene expression levels (n=9-10/concentration). Data in **(A, C-G)** were pooled from two independent experiments and analyzed with a two-tailed Student’s *t*-test, and the results are shown as mean ± SD. *p < 0.05, **p < 0.01, ***p < 0.001, ****p < 0.0001.

To verify that our findings using islets, *in vivo* and/or *ex vivo*, were indeed changes in β-cells, we tested the β-cell line, NIT-1 cells. We cultured NIT-1 cells, after the cells became confluent, in the presence of different concentrations of CP673451, a potent Pdgfr inhibitor (31), and solvent for 12 hours. The supernatants and NIT-1 cells were collected for insulin content and the NIT-1 cells were subject to Annexin V assay. Some NIT-1 cells were also prepared for RNA extraction (**Supplementary Figure S8**). In line with our *in vivo* findings in KO mice (**Figures 1E, F**), inhibition of Pdgfrα in NIT-1 β-cells resulted in reduced insulin secretion (**Figure 6A**). The reduction did not appear to be inhibitor dose dependent (**Figure 6A**). Also supporting our *ex vivo* finding in KO mice (**Figures 4D–F**), inhibiting Pdgfrα in NIT-1 β-cells led to a dose-dependent decrease in live cells (negative for Annexin 5 & 7AAD, **Figures 6B, C**) but increased apoptotic cells (**Figures 6B, D**). At the molecular level, we found that inhibition of Pdgfrα in NIT-1 β-cells led to decreased expression of *atf5* (**Figure 6E**), but increased *gadd45b* expression (**Figure 6F**), as well as reduced expression of *pi3k* (**Figure 6G**). Thus, the results from our *in vitro* investigation using NIT-1 β-cells with the Pdgfrα inhibitor validated our findings *in vivo* using mice with a β-cell targeted deletion of Pdgfrα. Taken together, using different experimental systems, we have revealed that Atf5 and Gadd45b are most likely the main players contributing to the impaired glucose metabolism seen in the KO mice.

## Discussion

**Figure 7:**
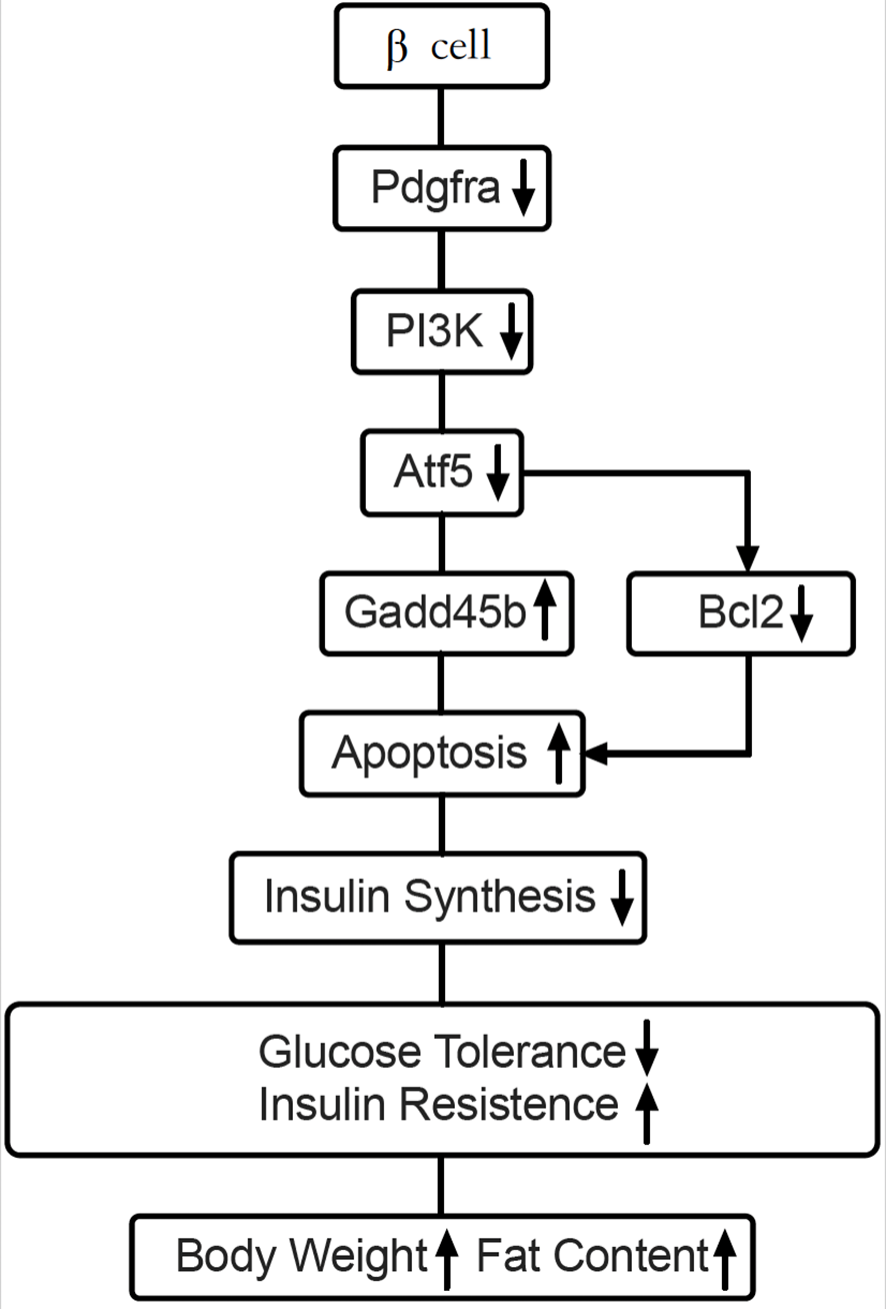
Working hypothesis: Pdgfrα regulates apoptosis through Atf5 and Gadd45b in β-cells. Our findings illustrate that Pdx-Cre driven Pdgfrα deficiency reduces Atf5 expression by down-regulating PI3K. Atf5 reduction leads to downstream Gadd45b upregulation, which results in apoptosis of pancreatic islet β-cells from C57BL/6 mice and decreased insulin synthesis. Atf5 could also regulate anti-apoptotic gene Bcl2. These pathways, are either enhanced or reduced, leading to the dysregulated glucose metabolism and obesity in C57BL/6 mice.

Pdgfrα is a widely expressed molecule that influences cell development and function. However, its role in insulin-producing β-cells was not previously fully understood. In this report, to investigate the role of Pdgfrα in β-cell function and glucose metabolism, we generated mice with β-cell-specific deletion of *Pdgfrα* using a *Pdgfrα* ^fl/fl^ and Pdx1Cre system, and made several novel discoveries. First, we demonstrated that *Pdgfrα-*deficient mice had dysregulated glucose metabolism, increased body weight with more adipose tissue, especially visceral fat and a higher ratio of body fat vs body weight. Second, β-cell-specific *Pdgfrα*-deficient mice had reduced insulin secretion but increased insulin resistance, which was more evident in the adult mice. Moreover, related to skeletal muscle inflammation, it is known that there is cross talk between adipose tissue and muscle and adipokines may attract inflammatory immune cells to skeletal muscle tissue. It is also possible that alpha-cells produce more glucagon, which increases blood glucose, further contributing to insulin resistance. The metabolic dysregulation and insulin resistance was exacerbated when the mice were on a high-fat diet. Third, β-cell specific *Pdgfrα*-deficient mice had reduced islet mass and number accompanied by an increase in apoptotic β-cells. Last, through transcriptome analysis of islets, we identified a marked reduction of *atf5*, a transcription factor for cell growth and survival, and significant upregulation of *gadd45b*, a key transcription factor in DNA damage and apoptosis, in β-cell-specific *Pdgfrα*-deficient islets. We further confirmed the transcriptomic results by inhibiting *Pdgfrα* in a β-cell line. Taken together, we have identified an important regulatory role for Pdgfrα in islet β-cell growth and/or survival, glucose metabolism, obesity and insulin resistance (**Figure 7**). Distinguishing between β-cell-intrinsic and systemic effects of *Pdgfrα* deletion remains critical to the interpretation of our phenotypic observations. Our comprehensive dataset strongly supports the conclusion that the observed metabolic dysregulation arises specifically from β-cell-specific Pdgfrα deficiency, rather than off-target or systemic alterations for the following reasons: ***a***, *In vitro* glucose-stimulated insulin secretion (GSIS) defects were observed in islets isolated from KO mice, which exhibited significantly reduced insulin release in response to high-glucose stimulation (25 mmol/L) compared to WT islets (**Figure 1E**). This defect was observed in a controlled *in vitro* environment, completely independent of systemic factors such as circulating hormones or immune cells, directly demonstrating that β-cell function is intrinsically impaired by Pdgfrα deletion. ***B***, We further analyzed Pdgfrα (CD140a) expression specifically in pancreatic β-cells (CD45^-^FluoZin^+^) of young (3–4 weeks of age) and adult (10–12 weeks of age) β-cell-specific Pdgfrα-depleted mice, to explore potential age-dependent dynamics. As anticipated, Pdgfrα protein levels were lower in β-cells from Pdgfrα-deficient (KO) mice relative to wild-type (WT) controls across both age groups. Notably, the magnitude of this Pdgfrα reduction was substantially greater in young mice. Consistent with this, the percentage of CD140a-positive β-cells exceeded 10% in young mice (in both WT and KO, with KO showing a far more dramatic decline) but dropped to less than 1% in adult mice, irrespective of genotype (**Supplementary Figure S1**). This age-related decrease in β-cell Pdgfrα expression aligns with prior observations that Pdgfrα contributes to age-dependent β-cell proliferation and expansion (16), further supporting a context-dependent role of Pdgfrα in regulating β-cell biology. No significant changes in Pdgfrα expression were detected in other systemic tissues (e.g., duodenum tissue and brain hypothalamus tissue). Pdgfrα expression was slightly reduced in duodenal tissue but higher in brain hypothalamic tissue, neither of which were statistically significant. ***c***, We demonstrated temporal alignment of β-cell defects with systemic phenotypes with a clear sequence of events: β-cell dysfunction emerged first (6–7 weeks of age), characterized by impaired glucose tolerance (**Figure 1C**) and reduced insulin secretion (**Figure 1E**). Systemic metabolic phenotypes—including insulin resistance (**Figure 1L**) and increased adiposity (**Figures 1N,O**)—only developed later (≥14 weeks of age). This temporal order indicates that systemic metabolic changes are secondary consequences of β-cell-intrinsic defects, rather than independent effects of Pdgfrα deletion.

Atf5 belongs to the activating transcription factor/cyclic adenosine monophosphate (cAMP) response element binding protein family of the basic leucine zipper (bZip) transcription factors (32). Atf5 regulates gene transcription through binding of different DNA regulatory elements such as cAMP response element (CRE), ATF5-specific response element (ARE), and the amino acid response element (AARE) (33, 34). Atf5 is critical for pancreatic islet β-cell survival and Atf5 deficiency decreases β-cell survival under stress conditions, including endoplasmic reticulum (ER) stress, cytokine, and oxidative stress (35). Our results connect Pdgfr*α* with Atf5, both of which are important for β-cell development and survival. Atf5 expression can be substantially reduced by PI3K inhibition (27). Moreover, PI3K mediates an efficient mechanism for promoting some tissue functions by Pdgfrα (29, 30). Our study supports the notion that the reduction of Atf5 and PI3K is associated with increased β-cell apoptosis and β-cell function in *Pdgfrα^fl/fl^Pdx1-Cre^+^* mice.

We also found, in this study, that growth arrest and DNA-damage-inducible beta (Gadd45b), a member of the stress response Gadd45 family associated with cell apoptosis and survival (36), is involved in the apoptosis of β-cells in our mouse model. Gadd45b transcription is regulated, at least in part, by Atf4, an important paralog of Atf5, which regulates β-cell survival during stress (35, 36). In endoplasmic reticulum (ER) stress, overexpression of Atf5 results in decreased ER stress-induced apoptosis, whereas knockdown of *atf5* by RNA interference increases ER stress-induced apoptosis. Moreover, Atf5 deficiency decreased β-cell survival under stress conditions, likely enhancing the susceptibility of β-cells to stress-induced apoptosis (35, 37). Also in ER stress, Gadd45b expression was increased by various ER stressors such as Brefeldin A, Tunicamycin, Thapsigargin and Cadmium (38). Therefore, we suggest that the increased apoptosis of β-cells in *Pdgfrα^fl/fl^Pdx1-Cre^+^* C57BL/6 mice is due to decreased Atf5 expression, reducing the capacity to respond to β-cell stress, and thus the expression of the downstream stress sensor Gadd45b increases, which leads to β-cell apoptosis.

Of various cellular stresses, endoplasmic reticulum (ER) stress is critical in obesity and type 2 diabetes, and it contributes to β-cell failure (5, 39). The ER of β-cells is the site for insulin synthesis, folding and processing. It maintains specialized complexes of quality-control systems, including chaperones and foldases, to ensure the homeostasis of a unique equilibrium between the cellular demand for insulin synthesis and the ER folding capacity, to promote insulin transport and maturation (39). In response to high level nutrients, β-cells enhance their overall speed of both proinsulin transcription and translation (40, 41). Failure of β-cell ER adaptive capacity results in activation of the unfolded protein response (UPR), which intersects with many different ER stress signaling pathways (5). Atf4 is at the core of ER stress, and an important paralog of Atf5, upregulates aminoacyl-tRNA synthetases to recover protein translation in β-cells during ER stress (42). Interestingly, a downstream target of Atf5 is Bcl-2, an anti-apoptotic molecule, which acts in an Atf5-specific response element (ARE)-dependent fashion and mediates the pro-survival function of Atf5 (23). Cellular stress most likely contributes to our findings of increased β-cell apoptosis and reduced insulin content and insulin release in the absence of Pdgfrα.

In summary, Atf5 binds to the Atf5-specific response element (ARE) in the Bcl-2 promoter, directly transactivating Bcl-2 expression to inhibit mitochondrial apoptotic pathways (23). While Atf5 does not directly bind to the Gadd45b promoter, its downregulation likely relieves a transcriptional repressive effect on Gadd45b. This is consistent with studies showing that the paralog of Atf5, Atf4, indirectly suppresses Gadd45b during endoplasmic reticulum (ER) stress (35, 36). Atf5 binds to amino acid response elements (AAREs) in the promoters of regulating translation-related genes involved in protein synthesis (33, 34), and its downregulation would impair the transcription of aminoacyl-tRNA synthetases, which are rate-limiting enzymes in insulin biosynthesis. In our model system, Pdgfrα deletion suppresses Atf5 expression in β-cells and impairs β-cell function and survival, which leads to higher fasting blood glucose that is more evident in adult mice or mice on a high fat diet. Increased blood glucose requires a high demand for proinsulin biosynthesis. However, suppressed Atf5 is not able to reverse ER stress causing reduction of aminoacyl-tRNA synthetase expression, followed by increased Gadd45b expression, decreased Bcl2 expression, apoptosis, and decreased insulin synthesis and secretion. As a result of suppressed Atf5, the rise of blood glucose and β-cell stress will be part of a vicious cycle, which induces β-cell apoptosis that is not mediated through the action of Fas and Caspase 9.

In defining these mechanisms, we are aware that there are some limitations in our study. Although we have confirmed our findings related to impaired islet β-cells in the *Pdgfrα^fl/fl^Pdx1-Cre+* C57BL/6 mice with a β-cell line through inhibiting Pdgfrα, it is not yet clear whether developmental factors contribute to the impaired β-cell function seen in the *Pdgfrα^fl/fl^Pdx1-Cre+* C57BL/6 mice. This requires further investigation, perhaps using an inducible model system with *Pdgfrα* deficiency in β-cells. We are aware that the Pdx1 gene can be expressed in the intestine (duodenum), albeit at a low level in adult mice. Although in a different study using Pdx1-Cre system, we could not detect Pdx1 expression in duodenum by qPCR, it is not known whether intestinal homeostasis is affected in our *Pdgfrα^fl/fl^ Pdx1-Cre+* C57BL/6 model system. Thus, this also requires further investigation. In addition, pancreatic islets provided the template for our RNA-sequencing, rather than purified β-cells, due to technical challenges in our model system. Future application of single-cell RNA-sequencing technology will give us better resolution and information about the β-cell signature in our model system. Alternatively, a β-cell reporter mouse system with β-cell deficiency in *Pdgfrα* could be generated. Last, but not least, it is not yet clear whether Aft5 and Gadd45b interact directly or indirectly. Despite these limitations listed above, our study has shown a novel pathway for Pdgfrα regulation of β-cell function and glucose metabolism.

Taken together, we have demonstrated that Pdgfrα regulates Atf5, Gadd45b, β-cell apoptosis and insulin biosynthesis in mice *in vivo* and in a β-cell line *in vitro*. Our findings provide insight into a novel molecular loop for pathological β-cell apoptosis and metabolic dysfunction. If this finding were validated in fresh human islets from healthy and type 2 diabetic donors, it would provide strong support to a novel approach for designing better preventive and/or therapeutic strategies in relation to β-cell apoptosis in diabetes and beyond.

## Data Availability

All materials and data generated and analyzed in this study are available to the public and scientific community upon request. The RNA-seq data were deposited to NCBI/GEO and the accession # is GSE3.
