# Supplementary material for: Pdgfrα deficiency in islet β-cells up-regulates apoptosis of beta-cells and disturbs glucose metabolism in B6 mice
Source: Front Endocrinol (Lausanne). 2025 Oct 29;16:1630979. doi: 10.3389/fendo.2025.1630979 (PMC12605365; doi:10.3389/fendo.2025.1630979)
Supplement: Supplementary file 1 [file DataSheet1.pdf]

Pdgfrα deficiency in islet β-cells up-regulates apoptosis of beta-cells and disturbs glucose metabolism in B6 mice

Luyao Zhang [1,2*,#], Yanpeng Xing[1,2*], Pai Wang[1,2], Jianlei Gu[3], Jian Peng[2], Juan Huang [2,4], James Alexander Pearson[2,5], Youjia Hu[2], Hongyu Zhao[3], F. Susan Wong[5], Li Wen[2,#]

1. Department of Gastrocolorectal Surgery, General surgery center, The First Hospital of Jilin University, Changchun, Jilin, 130021, China.

2. Section of Endocrinology, Department of Internal Medicine, School of Medicine, Yale University, New Haven, Connecticut, 06520, USA.

3. Department of Biostatistics, Yale School of Public Health, New Haven, Connecticut, 06510, USA.

4. National Clinical Research Center for Metabolic Diseases, Key Laboratory of Diabetes Immunology (Central South University), Ministry of Education; and Department of Metabolism and Endocrinology, The Second Xiangya Hospital, Central South University, Changsha, Hunan, 410011, China.

5. Division of Infection and Immunity, School of Medicine, Cardiff University, Cardiff, CF14 4XN UK.

[*]These authors contributed equally: Luyao Zhang, Yanpeng Xing.

# Correspondence: Li Wen, S141, TAC 300, Cedar Street, New Haven, CT 06520-8103, USA. *Email:* li.wen@yale.edu; and Luyao Zhang, 1st Xinmin Street, Chaoyang District, Changchun, Jilin Province, China. *Email*: luyao@jlu.edu.cn

Figure S1.

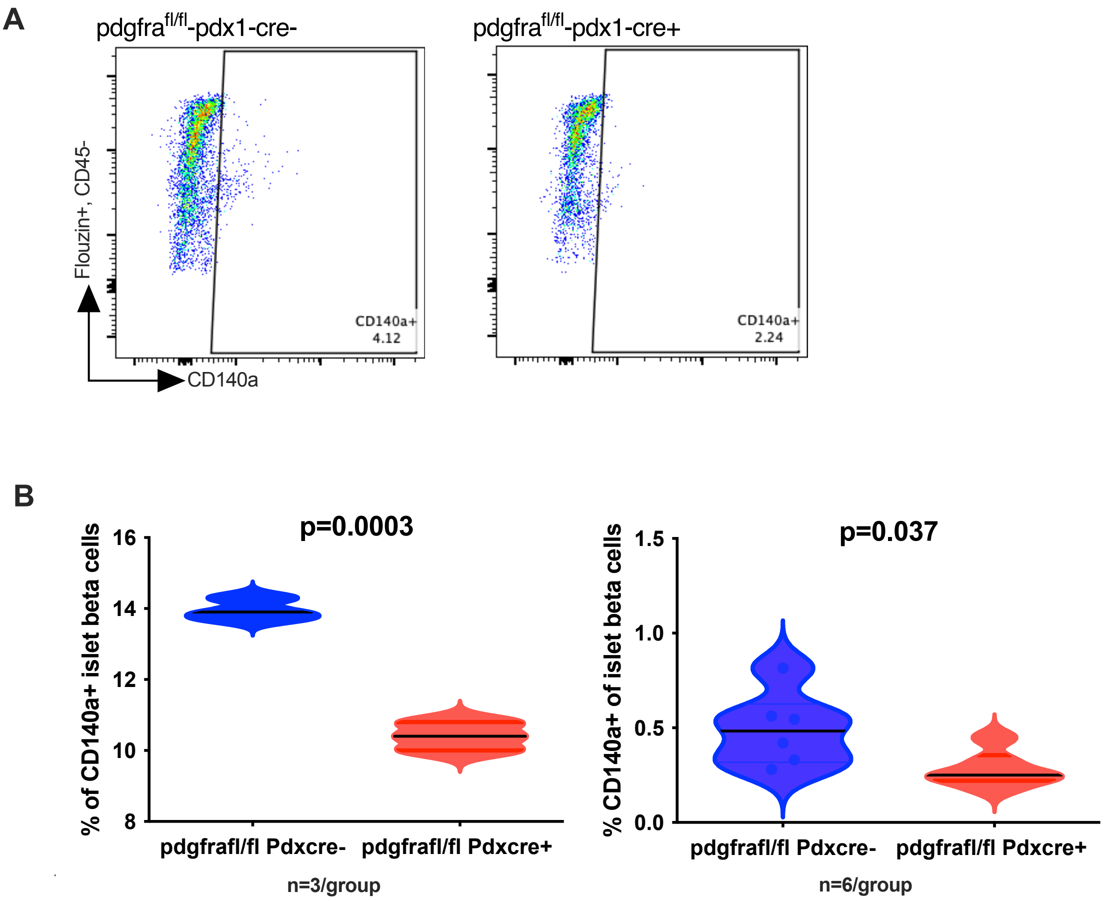

Figure S1. Reduction of Pdgfrα expression in β-cells from *Pdgfra*$^{fl/fl}$-*Pdx1-Cre*$^{+}$ C57BL/6 mice. The protein expression of Pdgfrα on islet β-cells was assessed by flow cytometry. Islets were isolated from *Pdgfra*$^{fl/fl}$-*Pdx1-Cre*$^{+}$ and *Pdgfra*$^{fl/fl}$-*Pdx1-Cre*$^{-}$ control mice. Dissociated single cells were stained with CD45 (immune cell marker), FluoZin (β-cell marker) and CD140a (Pdgfrα). β-cells were identified as FluoZin+CD45- after gating out any remaining cell aggregates. As FluoZin is shown by intracellular staining, no live/dead cell marker was used in the staining. **(A)** Representative gated flow plots. **(B)** Quantification of the flow cytometry analysis. β-cell Pdgfrα (CD140a) expression in young (3-4 wks of age) and adult (10-12 wks) mice. The β-cells from *Pdgfra*$^{fl/fl}$-*Pdx1-Cre*$^{+}$ mice express significantly lower Pdgfrα protein (CD140a) compared with those from control mice. Two-tailed Student's *t*-test was used for statistical analysis, and the results are represented as mean ± SD. P<0.05 was considered as statistically significant.

Figure S2.

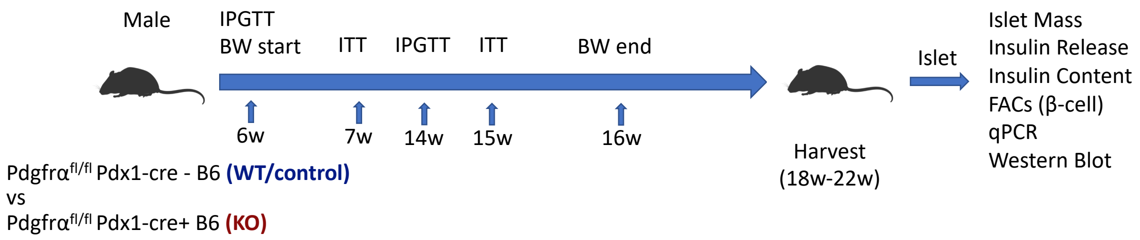

Figure S2. A schematic of the experimental design in mice on normal diet. The weekly body weight (BW) measurement was started when the mice were 6 weeks of age to 16 weeks. IPGTT was assessed at 6 and 14 weeks of age, respectively. ITT was assessed at 7 and 15 weeks of age, respectively. The mice were terminated at 18 to 22 weeks of age, and the experiments listed were performed.

Figure S3.

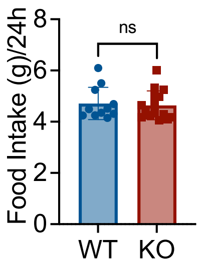

Figure S3. Food intake of the KO mice and the control mice.

Figure S4.

Figure S4. Islet structure and cell composition of the KO and control mice. Fresh ex-vivo pancreases were formalin-fixed and paraffin-embedded followed by cutting into 10µm sections. Tissue sections were stained with H&E (A), anti-insulin and anti-ki67 (B) or anti-glucagon (C). Light microsopy of sections from a representative mouse in each group (n=3-6 mice/group) with amplification of 40X for A, 100X for B and C are shown.

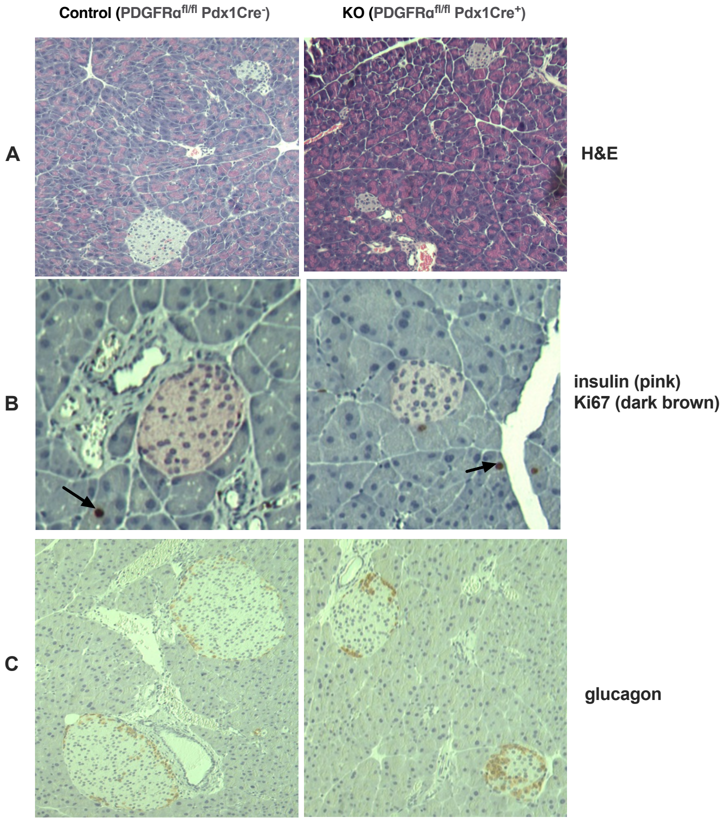

Figure S5.

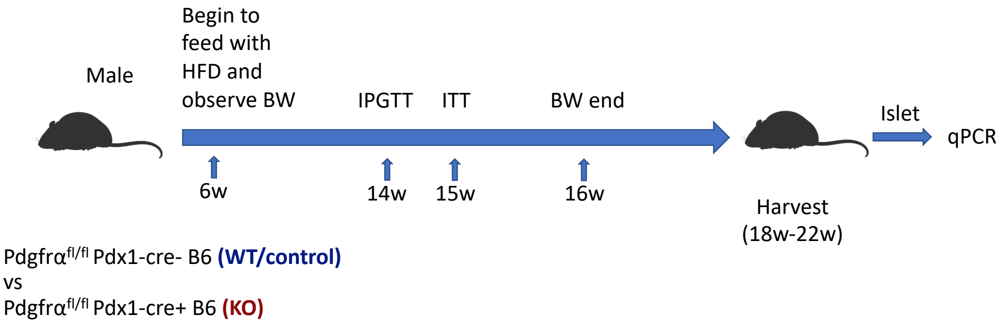

Figure S5. A schematic of the experimental design for the mice fed with high fat diet (HFD). HFD started when the mice were 6 weeks old, and their BW was measured weekly to 16 weeks of age. IPGTT was performed at 14 weeks of age (8 weeks after HFD) and ITT was assessed at 15 weeks of age. The mice were terminated at 18 to 22 weeks of age followed by *in vitro* studies.

Figure S6.

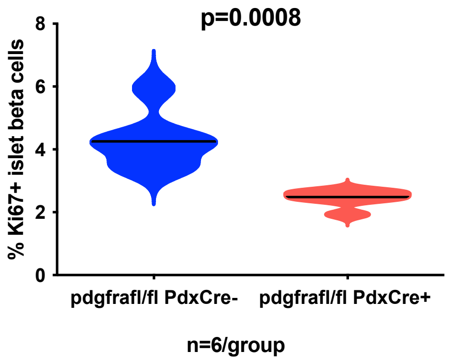

Figure S6. Reduction of Ki67 positive β-cells from *Pdgfra*$^{fl/fl}$-*Pdx1-Cre*$^{+}$ C57BL/6 mice. Islet β-cell proliferation shown by staining dispersed islet cells with Ki67, costained with anti-mouse CD45 and FluoZin and and analyzed by flow cytometry. After gating on islet β-cells (CD45-FluoZin+), we analyzed the proportion of Ki67 positive (Ki67+) cells. The β-cells from *Pdgfra*$^{fl/fl}$-*Pdx1-Cre*$^{+}$ mice express significantly lower Ki67 than those of the control mice. Two-tailed Student's *t*-test was used for statistical analysis, and the results are represented as mean ± SD. $P<0.05$ was considered as statistically significant.

Figure S7

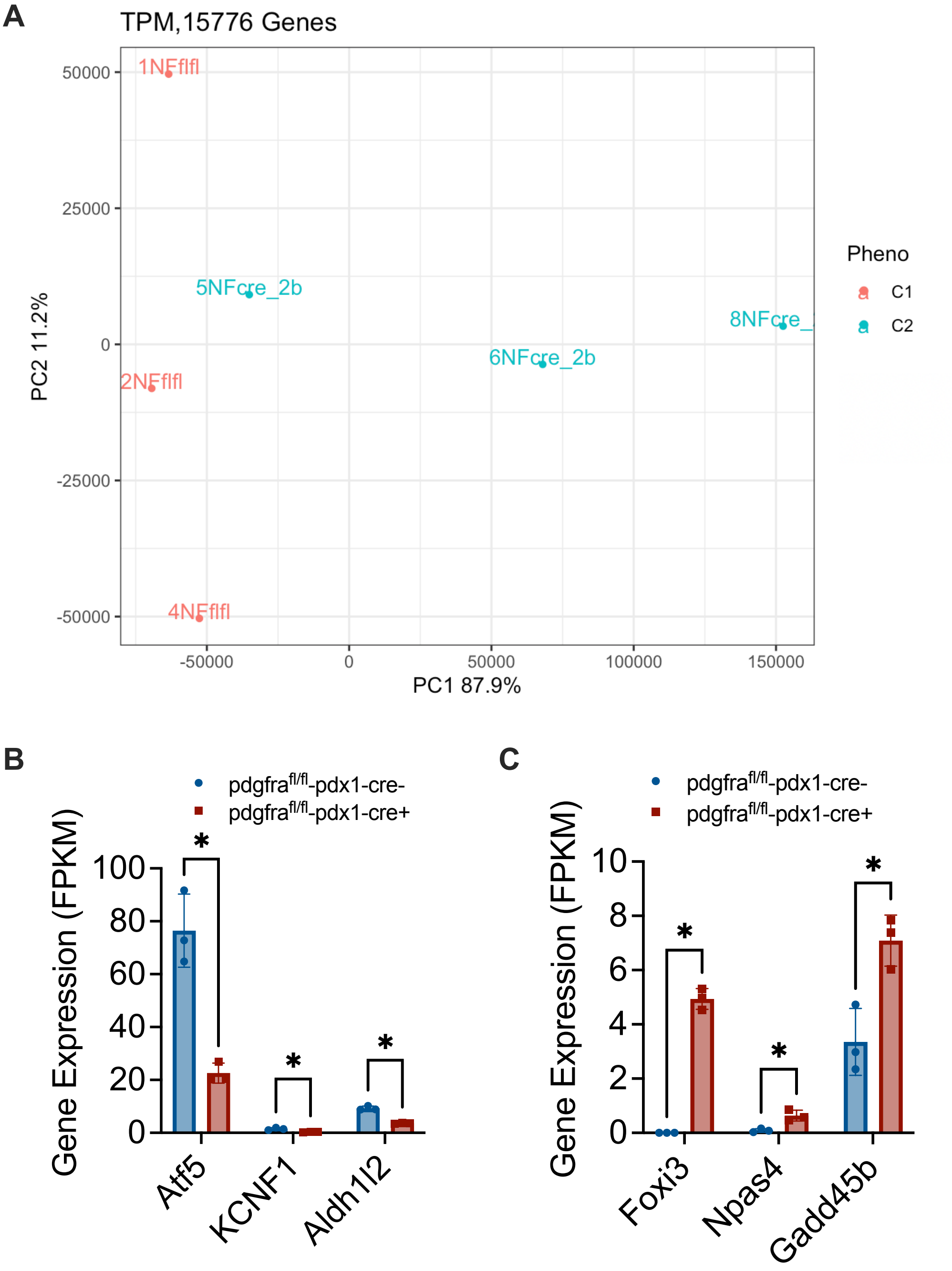

Figure S7 Analysis of RNA sequences of islets from *Pdgfra*$^{fl/fl}$-*Pdx1-Cre*+ C57BL/6 vs *Pdgfra*$^{fl/fl}$-*Pdx1-Cre*+ C57BL/6 mice (n=3/group). Principal component analysis (PCA) plot of the RNA-sequencing data. Red: islets from *Pdgfra*$^{fl/fl}$-*Pdx1-Cre*$^{+}$ C57BL/6 mice, blue: islets from *Pdgfra*$^{fl/fl}$-*Pdx1-Cre*$^{-}$ C57BL/6 mice. The two groups are separated significantly, indicating that the transcriptomes of the islets from the two groups are very different.

Figure S8.

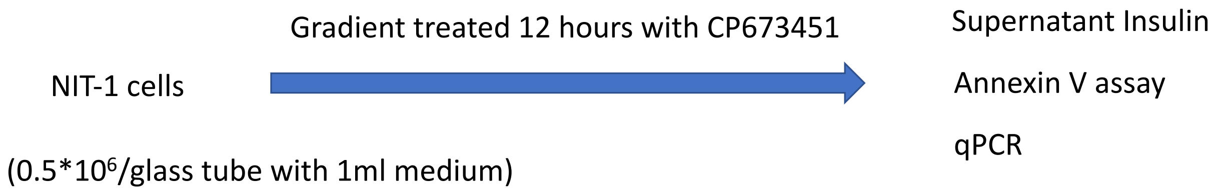

Figure S8. Schematic of the experimental design using NIT-1 cells. When NIT-1 cells became confluent in the cell culture flask, different concentrations of the Pdgfr inhibitor, CP673451(inhibiting both Pdgfr-β and -α), was added to the culture for 12 hours. Culture supernatants were collected for insulin measurement. NIT-1 cells were harvested for flow cytometry and qPCR assays.

Table S1. Top 8 clusters with their representative enriched terms (one per cluster).

GO	Category	Description	Count	%	Log10(P)	Log10(Q)
GO:0006418	GO Biological Processes	tRNA aminoacylation for protein translation	6	14.29	-9.74	-5.71
R-MMU-196757	Reactome Gene Sets	Metabolism of folate and prerinse	3	7.14	-5.40	-1.98
GO:1901607	GO Biological Processes	alpha-amino acid biosynthetic process	3	7.14	-3.73	-0.61
GO:0048538	GO Biological Processes	thymus development	3	7.14	-3.68	-0.60
GO:0098739	GO Biological Processes	import across plasma membrane	4	9.52	-3.56	-0.55
GO:0050885	GO Biological Processes	neuromuscular process controlling balance	3	7.14	-3.42	-0.44
GO:0051952	GO Biological Processes	regulation of amine transport	3	7.14	-2.72	0.00
GO:0032434	GO Biological Processes	regulation of proteasomal ubiquitin-dependent protein catabolic process	3	7.14	-2.64	0.00
GO:0006417	GO Biological Processes	regulation of translation	4	9.52	-2.23	0.00
GO:0030900	GO Biological Processes	forebrain development	4	9.52	-2.01	0.00

For the given gene list, pathway and process enrichment analysis has been carried out with the following ontology sources: GO Biological Processes, KEGG Pathway, Reactome Gene Sets, CORUM and WikiPathways. All genes in the genome have been used as the enrichment background. Terms with a p-value < 0.01, a minimum count of 3, and an enrichment factor > 1.5 are collected and grouped into clusters based on their membership similarities. The most statistically significant term within a cluster was chosen to represent the cluster.

Table S2. Primer information

Genes	Primers	Sequence
Atf5	Forward	5′- AATTGAGGTGTATAAGGCCCG -3′
	Reverse	5′- GGATAGGAAAGTGGAATGGAGG -3′
Gadd45b	Forward	5′- GTTCTGCTGCGACAATGACA -3′
	Reverse	5′- TTGGCTTTTCCAGGAATCTG -3′
Bcl2	Forward	5′- GCTGGGGATGACTTCTCTCG -3′
	Reverse	5′- CCACAATCCTCCCCCAGTTC -3′
Fas	Forward	5′- CTGTCCTGCCTCTGGTGCTT -3′
	Reverse	5′- AGCAAAATGGGCCTCCTTGA -3′
Caspase9	Forward	5′- TCCTGGTACATCGAGACCTTG -3′
	Reverse	5′- AAGTCCCTTTCGCAGAAACAG -3′
PI3K	Forward	5′- ATCATGCAAATCCAGTGCAA -3′
	Reverse	5′- CAGCTGTCCGTCATCTTTCA -3′
Gapdh	Forward	5′- GGGGTCGTTGATGGCAACA-3′
	Reverse	5′- TGTAGACCATGTAGTTGAGGTCA-3′

Table S3. List of primary and secondary antibodies used in this study.

Target	Host Species	Type	Application	Source
CD45	Rat	Monoclonal	FC	Biolegend
CD140a	Rat	Monoclonal	FC	Biolegend
Atf5	Rabbit	Polyclonal	WB	Invitrogen
Gadd45b	Rabbit	Polyclonal	WB	Bioss
PI3K p85	Rabbit	Monoclonal	WB	Cell Signaling
p-PI3K p85/55	Rabbit	polyclonal	WB	Cell Signaling
Gapdh	Rabbit	monoclonal	WB	Cell Signaling
Rabbit IgG	Donkey	polyclonal	WB	Biolegend
